# High-efficiency CRISPR/Cas9 multiplex gene editing using the glycine tRNA-processing system-based strategy in maize

**DOI:** 10.1186/s12896-016-0289-2

**Published:** 2016-08-11

**Authors:** Weiwei Qi, Tong Zhu, Zhongrui Tian, Chaobin Li, Wei Zhang, Rentao Song

**Affiliations:** 1 Shanghai Key Laboratory of Bio-Energy Crops, School of Life Sciences, Shanghai University, 333 Nanchen Road, Shanghai, 200444 China; 2 National Maize Improvement Center of China, China Agricultural University, Beijing, 100193 China; 3 Coordinated Crop Biology Research Center (CBRC), Beijing, 100193 China

**Keywords:** CRISPR/Cas9, Maize, Multiplex gene editing, tRNA-processing

## Abstract

**Background:**

CRISPR/Cas9 genome editing strategy has been applied to a variety of species and the tRNA-processing system has been used to compact multiple gRNAs into one synthetic gene for manipulating multiple genes in rice.

**Results:**

We optimized and introduced the multiplex gene editing strategy based on the tRNA-processing system into maize. Maize glycine-tRNA was selected to design multiple tRNA-gRNA units for the simultaneous production of numerous gRNAs under the control of one maize U6 promoter. We designed three gRNAs for simplex editing and three multiple tRNA-gRNA units for multiplex editing. The results indicate that this system not only increased the number of targeted sites but also enhanced mutagenesis efficiency in maize. Additionally, we propose an advanced sequence selection of gRNA spacers for relatively more efficient and accurate chromosomal fragment deletion, which is important for complete abolishment of gene function especially long non-coding RNAs (lncRNAs). Our results also indicated that up to four tRNA-gRNA units in one expression cassette design can still work in maize.

**Conclusions:**

The examples reported here demonstrate the utility of the tRNA-processing system-based strategy as an efficient multiplex genome editing tool to enhance maize genetic research and breeding.

**Electronic supplementary material:**

The online version of this article (doi:10.1186/s12896-016-0289-2) contains supplementary material, which is available to authorized users.

## Background

Mutants are critical in genetic research for the study of gene function, and gene editing technologies can efficiently create mutations in targeted genes. The clustered regularly interspersed short palindromic repeat (CRISPR)/CRISPR-associated protein (Cas) system has evolved from studies of the defense systems of bacteria to a newly established gene editing tool [1]. The CRISPR/Cas9 system is derived from *Streptococcus pyogenes* and possesses a protospacer adjacent motif (PAM) recognition sequence [2, 3]. The Cas9 gene and a 20-bp guide RNA (gRNA) that is complementary to the DNA site being targeted for mutation need to be transformed into the target organism to create a gene disruption. The CRISPR/Cas9 system has been demonstrated for efficient gene disruption in multiple organisms, including bacteria [1], yeast [4], zebrafish [5], fruit flies [6], human cells [7] and plants [8]. In plants, the CRISPR/Cas9 system has been effectively applied in many species, including *Arabidopsis thaliana*, *Citrus sinensis*, *Nicotiana tabacum*, *Oryza sativa*, *Solanum lycopersicum*, *Sorghum bicolor*, *Triticum aestivum* [9], *Zea mays* [10–13], and *Glycine max* [14]. For highly efficient gene modification, the CRISPR/Cas9 vector construction strategy should always be optimized for the usage in specific organism.

Gene editing tools with the capability to manipulate multiple targets are of great value. The CRISPR/Cas9 system is a promising tool for this purpose. Multiplex gene editing can be achieved by expressing Cas9 along with multiple gRNAs, each targeting different sites. Conventional delivery methods involve creating gene constructs containing multiple gRNA expressing cassettes for multiplex gene editing in one plasmid or using multiple plasmids [15–20]. Due to the limitations of the delivery method and plasmid capacity, compacting multiple gRNAs into one synthetic gene would be an advanced strategy. Xie et al. [21] demonstrated that multiple gRNAs could be efficiently produced from a single synthetic gene using a tRNA–gRNA architecture that allows for the precise excision of transcripts *in vivo* by the endogenous RNases, RNase P and Z, in rice. This strategy could be broadly used to generate multiplex gene editing in both monocot and dicot plants after specific optimization, because the tRNA-processing system exists in virtually all organisms.

Although the CRISPR/Cas9 system showed high efficiency for genome modification, it did not always create strong mutation with complete abolishment of gene function especially for long non-coding RNAs (lncRNAs). Chromosomal fragment deletion between target sites can be archived with multiplex gene editing strategy and constitutively improves the mutation degree [21]. As the process of gene modification by CRIPSR/Cas9 system depends on the gRNA to search and target to the specific site and the target sites meeting the requirements for targeting cannot always be targeted at the same time [5], the selection of gRNA spacers is essential for high efficient chromosomal fragment deletion.

Maize (*Zea mays*) is one of the most important cereal crops in the world. Here, we report our specific vector construction, sequence design and editing results of using the multiplex gene editing strategy based on the tRNA-processing system in maize. The design of the vectors was optimized for the usage in maize. We found the tRNA-processing system-based method improves the efficiency of CRISPR/Cas9 editing in maize and proposed an advanced gRNA selection strategy for chromosomal fragment deletion purpose.

## Methods

### Plant materials

The HiII maize parental lines PA and PB were initially obtained from the Maize Genetics Cooperation Stock Center and maintained in the lab. The transgenic lines were generated in the lab. Maize plants were cultivated in the experimental field, green house or growth chambers at the campus of Shanghai University.

### Construction of plant transformation vectors

The maize U6 promoter (U6p) and U6 terminator (U6t) were amplified using gene-specific primers (see Additional file 1: Table S1 for primer sequences) and cloned into the *Pst*I site of the pCAMBIA3301 vector with the maize codon optimized *Cas9* gene (from Jinsheng Lai’s lab) [12]. gRNAs designed for simplex editing and tRNA-gRNA units (TGUs) designed for multiplex editing were synthesized by Generey (Generey.com) and cloned into the *Psi*I and *Xba*I sites between the U6 promoter and U6 terminator. The constructed plasmids, pCAMBIA3301 with *UBQp: Cas9* and *U6p: gRNA* or *U6p: TGUs*, were used for *Agrobacterium*-mediated maize transformation.

### *Agrobacterium*-mediated transformation of immature maize embryos

*Agrobacterium*-mediated maize transformation was carried out according to Frame et al., [22]. Between 11 and 21 independent transgenic lines were generated for each transformation and genotyped with BAR specific primers (see Additional file 1: Table S1 for primer sequences).

### Genomic DNA extraction and PCR/Sequencing assay

For each BAR-positive transgenic line, three individual tissue samples were used to extract genomic DNA. Maize genomic DNA was extracted with the hexadecyltrimethylammonium bromide method [23]. Target regions were amplified with specific primers pairs flanking the designed target sites (see Additional file 1: Table S1 for primer sequences) using KOD DNA polymerase (Toyobo) to detect mutagenesis at the desired sites. The PCR product was separated on a 1 % agarose gel and stained using ethidium bromide. The stained gels were imaged using the Gel Doc XRS system (Bio-Rad). Selected PCR products were cloned into the pGEM-T Easy Vector (Promega) for DNA sequencing. For PCR product of each tissue sample, twenty clones were sequenced to detect stable editing.

### Zein extraction and quantification

Mature kernels of either WT or MADS/Cas9 line 21 were collected from well-filled ears. Zeins were extracted from 50 mg of dried endosperm flour according to previously described methods [24]. Extracted proteins were measured using a bicinchoninic acid protein assay kit (Pierce) according to the instructions. Measurements of all samples were replicated three times. SDS-PAGE was performed on 12 % polyacrylamide gels and visualized by staining with Comassie brilliant blue (Dingguo).

## Results

### Strategy to engineer simplex editing and multiplex editing based on the tRNA-processing system in maize

**Fig. 1:**
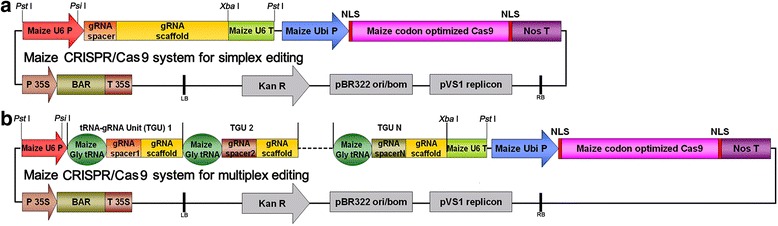
Simplex and multiplex editing vector construction strategy. **a** & **b** Structure of simplex and multiplex editing binary vector based on pCAMBIA3301. P, promoter; T, terminator; Ubi P, ubiquitin promoter; NLS, nuclear localization sequence; Nos T, nopaline synthase terminator; BAR, phosphinothricin R; Kan R, kanamycin resistance gene; LB, left border; RB, right border; TGU, tRNA-gRNA unit

A maize codon optimized Cas9 driven by the maize ubiquitin (UBQ) promoter was inserted into pCAMBIA3301 (see Methods) to construct two binary CRISPR/Cas9 vectors for either simplex editing or multiplex editing (Fig. 1). These two vectors both contain the *BAR* gene as a plant-selectable marker.

**Fig. 2:**
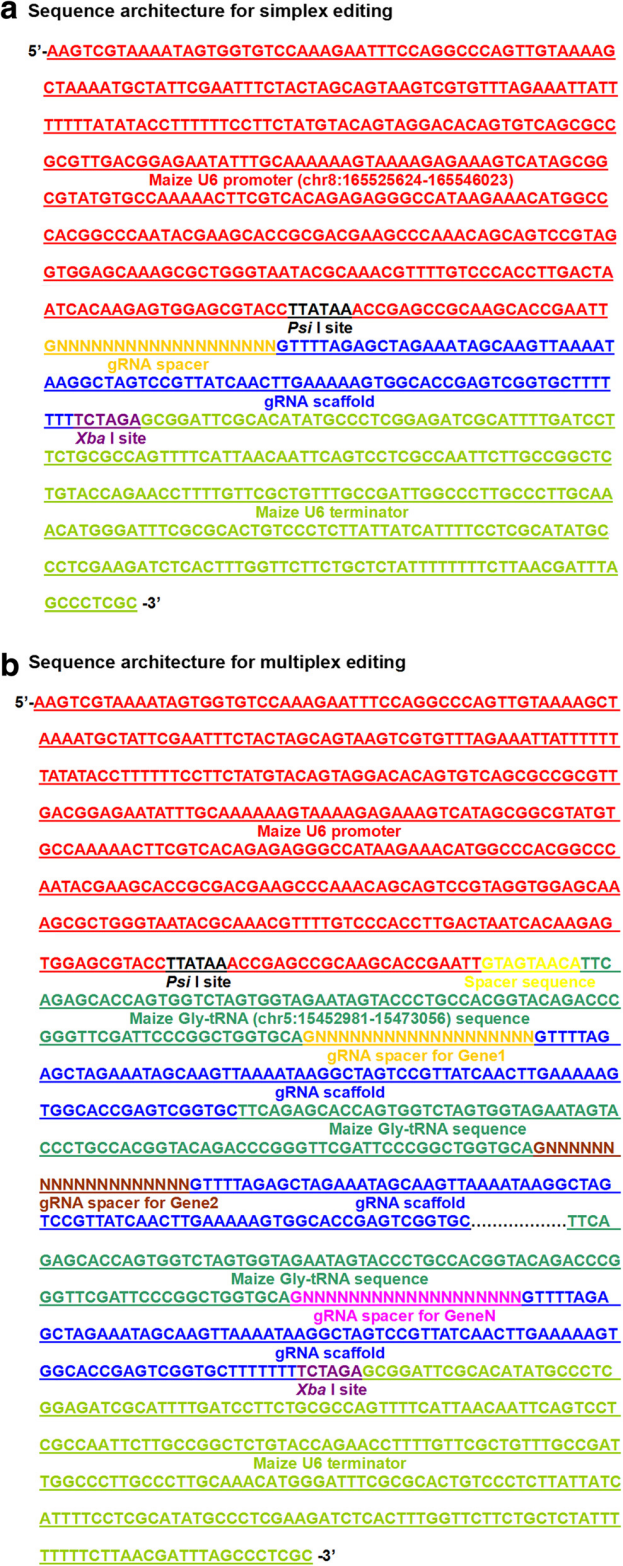
gRNA for simplex editing and the tRNA-gRNA units for multiplex editing sequence design strategy. **a** & **b** The specific sequence of the core elements of the simplex editing and multiplex editing vectors designed for the usage in maize, which can be directly used for broad gene knock-out in further maize genetics and maize breeding studies

For the simplex editing vector, we selected and cloned a small nuclear U6 RNA promoter from maize (U6p, Chr 8: 165525624-165548023) and the corresponding U6 terminator (U6t) to facilitate the expression of the gRNA cassette in the CRISPR/Cas9 construct. The gRNA with the target sequence (gRNA spacer) is transcribed from the U6 promoter with a definite transcription initiation site G nucleotide [15]. Therefore, target sequences are commonly selected for the U6 promoter by searching for 5′-GN (19) NGG motifs (NGG: protospacer adjacent motif, PAM). It was reported that the gRNA spacer with extended nucleotides at the 5′ end, derived from the vector ligation site, could also guide genome editing in plants [25]. It is unclear whether this kind of gRNA spacer affects the editing efficiency. Therefore, we found a *Psi*I restriction site within the U6p to certify that the selected gRNA sequence directly followed the U6p without adding additional nucleotides at the 5′ end (Fig. 2a).

For the multiplex editing vector, we compacted a cluster of gRNAs with different spacers into one polycistronic gene to simultaneously produce multiple gRNAs from one primary transcript. Xie et al. [21] have proposed that the tRNA precursors, pre-tRNAs, are cleaved at specific sites in eukaryotes by RNase P and RNase Z to remove extraneous 5′ and 3′ sequences. The tRNA-processing system is used as an intrinsic mechanism to produce different small RNAs, for example, small nucleolar RNA (snoRNA), from a single polycistronic gene. They successfully obtained multiplex editing using multiple gRNAs produced from a single synthetic gene employing the tRNA–gRNA architecture in rice. We designed multiple tRNA-gRNA units (TGUs) for the simultaneous production of numerous gRNAs to utilize the endogenous tRNA-processing system-based strategy for multiplex editing in maize (Fig. 1b). We selected the maize glycine-tRNA (Chr 5: 15452981-15473056) for the construction of the TGUs, and a spacer sequence starting with a G nucleotide was inserted between the U6p and the first glycine-tRNA to certify the transcription initiation with G nucleotide (Fig. 2b). The multiple TGUs (MTs) consisted of tandem repeats of tRNA-gRNA and would be transcribed under the control of the U6p. The resulting gRNAs would then direct Cas9 to multiple target sites for genome editing.

### Effective and efficient multiplex editing in stable transgenic maize via the tRNA-processing system-based strategy

To explore the efficiency of genome modification by our multiplex editing strategy, we synthesized three gRNAs for simplex editing and three MTs for multiplex editing.

**Fig. 3:**
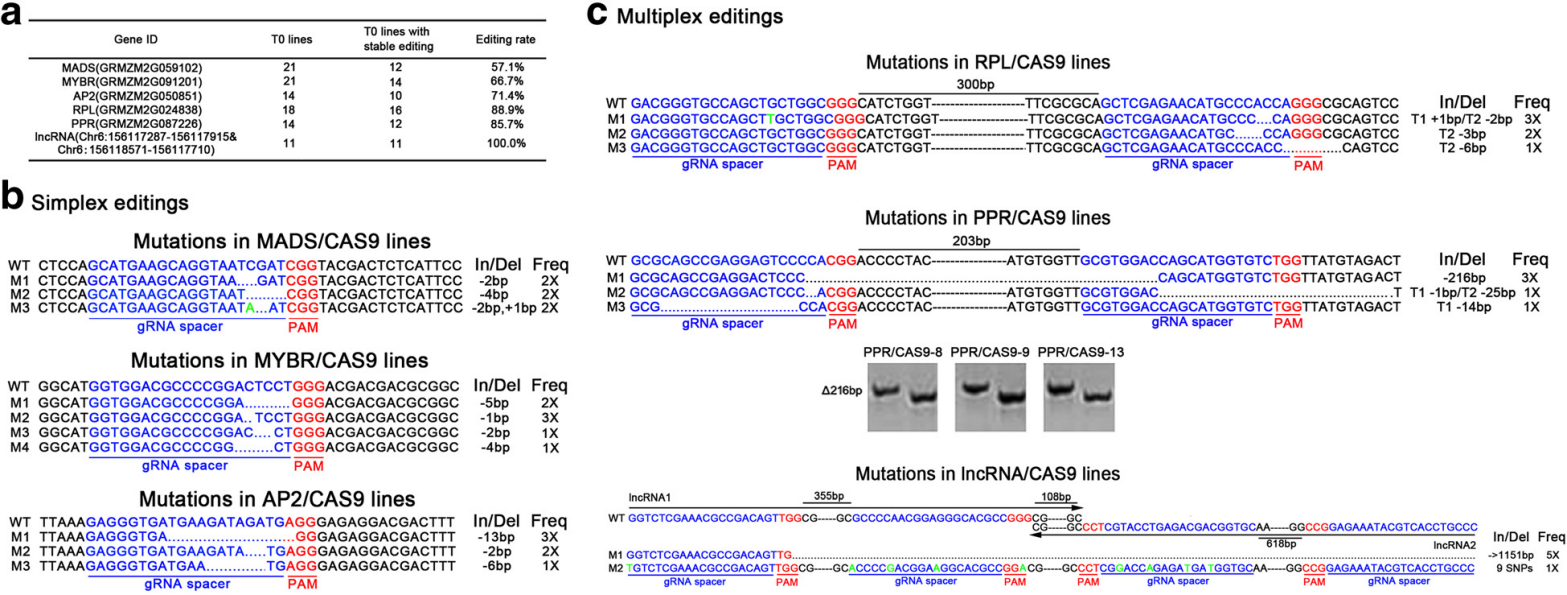
Simultaneous sequence editing results of simplex and multiplex editing. **a** Targeted mutation efficiency of simplex and multiplex edited plants. Editing rate showed the percentage of T0 transgenic lines with stable heritable editing within all T0 lines. **b** The DNA sequence of the simplex edited genes is provided. The 20-bp gRNA spacer sequence for the Cas9/gRNA complex is in blue, and the PAM site is in red. Deleted nucleotides are depicted as dots, and inserted nucleotides are shown in green. The lengths of the insertions and/or deletions (In/Del) and the frequencies (Freq) are presented. WT, wild type; M1, mutant1. **c** The DNA sequence of the multiplex edited genes is provided. The 20-bp gRNA spacer sequence for the Cas9/gRNA complex is in blue, and the PAM site is in red. Deleted nucleotides are depicted as dots, and inserted nucleotides are shown in green. The lengths of the insertions and/or deletions (In/Del) and the frequencies (Freq.) are presented. WT, wild type; M1, mutant1; T1, target1. Gel pictures show PCR products amplified from PPR/CAS9-8, 9 and −13 lines. Left lane, WT; right lane, mutant

The gRNAs for simplex editing target three transcription factors: a maize *MADS* gene (GRMZM2G059102), a maize *MYBR* gene (GRMZM2G091201), and a maize *AP2* gene (GRMZM2G050851). The *MADS* gene and the *MYBR* gene were both reported to be related to the maize endosperm-specific core transcription factor Opaque2 (Fig. 3a) [26, 27]. The GN (19) NGG gRNA spacer sequence selected for the targeting of *MADS* was at the 194 bp of its open reading frame (ORF). The gRNA spacer sequences for *MYBR* and *AP2* were at the 237 and 33 bp of the ORFs, respectively (Fig. 3b).

The gRNA-targeted genes for multiplex editing were a maize *RPL* gene (GRMZM2G024838), a maize *PPR* gene (GRMZM2G087226), and two reverse overlapping maize long non-coding RNAs (lncRNAs, Chr6:156117287-156117915 & Chr6:156118571-156117710) (Fig. 3a). *RPL* and *PPR* may be important for plant development in maize. The two lncRNAs were reported to be regulated by Opaque2 [26] and overlapped in opposite orientations in the maize genome. *RPL* and *PPR* were both targeted at two sites with a single 2 TGU construction. The two reverse overlapping lncRNAs were targeted with a single 4 TGU construction that included two sites for lncRNA1 and two sites for lncRNA2. The two GN (19) NGG sequences selected for targeting *RPL* were at 94 and 417 bp in its ORF, and the gRNA spacer sequences for *PPR* targeted 327 and 553 bp in its ORF. The two GN (19) NGG sequences selected for targeting lncRNA1 were at 62 and 440 bp, and gRNA spacer sequences targeting lncRNA2 were at the 52 and 693 bp (Fig. 3c).

We used conventional *Agrobacterium*-mediated transformation to produce the stable transgenic lines for the six constructs and evaluated the efficacy of our simplex and multiplex editing system. Twenty-one independent transgenic lines were generated for *MADS* and *MYBR*. Fourteen independent transgenic lines were generated for *AP2* and *PPR*, 18 independent transgenic lines were generated for *RPL*, and 11 independent transgenic lines were generated for the lncRNAs (Fig. 3a).

Mutagenesis frequency was examined in the T0 generation. Inheritance of the edited sites in T0 transgenic lines was desired for maize genetics and breeding researches and stable editing throughout the whole transgenic plant is heritable. For each BAR-positive transgenic line, three individual tissue samples were used to extract genomic DNA. Target regions were amplified with specific primers pairs flanking the designed target sites. The PCR product was cloned into the pGEM-T Easy Vector. For the three tissue samples of each transgenic line, twenty clones for PCR product of each tissue sample were sequenced to detect stable editing.

In the T0 generation of the MADS/CAS9 plants, 57.1 % (12 lines) carried stable editing including In/Dels and SNPs. The MADS/CAS9-21 transgenic plant had a biallelic mutation. We found stable mutations in 66.7 % (14 lines) of the T0 MYBR/CAS9 plants, and 71.4 % (10 lines) of the AP2/CAS9 T0 lines were mutants (Fig. 3a and b). Higher mutagenesis efficiency was achieved in the T0 generation of the multiplex editing lines than in the simplex editing lines. In the RPL/CAS9 plants, 88.9 % (16 lines) of T0 lines had stable mutations. However, the chromosomal-fragment deletion between two targets that can be achieved by the tRNA-processing system as reported by Xie et al. [21] was not detected in the RPL/CAS9 plants. This indicates that the MTs do not always operate on both targets simultaneously. The PPR/CAS9 plants had stable mutations in 85.7 % (12 lines) of T0 lines. The chromosomal-fragment deletion between target1 and target2 was detected in the PPR/CAS9-8, 9 and 13 T0 lines (Fig. 3c). Interestingly, these three lines also carry biallelic mutations. For the lncRNAs targeted from a single 4 TGU construct that included two sites for lncRNA1 and two sites for lncRNA2, 100 % (11 lines) of T0 lines had mutations. Surprisingly, a large region chromosomal-fragment deletion (about 2 kb) beyond the sequence between targets was detected in several transgenic lines (Fig. 3c), and there were only SNPs at target sites in other transgenic lines. Our data also demonstrated that the tRNA-processing system for multiplex editing not only increased the targeted sites but also significantly enhanced mutagenesis efficiency in maize (*p*-value = 0.021).

### Generation of phenotypic mutants

The biallelic transgenic line MADS/CAS9-21 for *MADS* gene and transgenic lines PPR/CAS9-9 and −13 for *PPR* gene were selected for further phenotypic analysis.

**Fig. 4:**
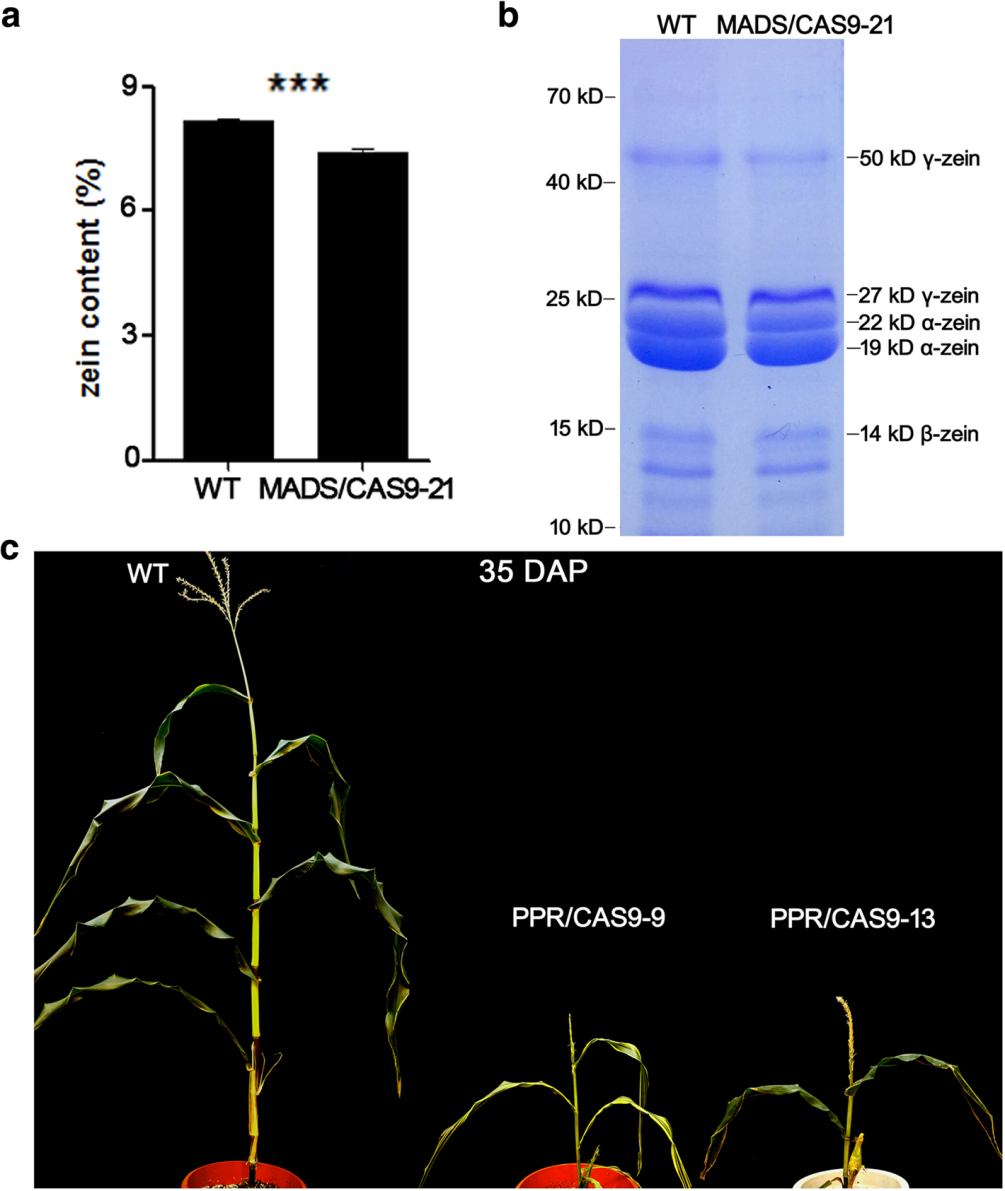
Phenotypic variations of simplex and multiplex editing mutants generated from the CRISPR/Cas9 targeting. **a** Comparison of zein protein contents from WT and MADS/CAS9 line 21 mature endosperm. The measurements were done on per 50 mg of dried endosperm. Error bars represent SD (*n* = 3) (***P < 0.001, Student’s t test). **b** SDS-PAGE detection of zein accumulation in WT and MADS/CAS9 line 21 mature endosperm. **c** Mature (35 DAP) WT and T0 transgenic maize plants of the mutant lines PPR/CAS9-9 and −13

Zeins are the most abundant storage proteins in maize kernels, and are encoded by different classes of genes. MADS (GRMZM2G059102) was reported to interact with Opaque2 and activate zein gene promoters. In *MADS RNAi* kernels, the expression of the 22-kD α-zein genes, the 19-kD α-zein genes and the 50-kD γ-zein gene decreased. Relative differences in these zein protein contents can be observed in *MADS RNAi* kernels [27]. Quantitative analysis showed that zeins were significantly decreased (12.5 %) in MADS/CAS9-21 kernels (Fig. 4a). We also observed differences in relative contents of the 22-kD α-zein, the 19-kD α-zein and the 50-kD γ-zein proteins between the wild type and MADS/CAS9-21 kernels through SDS-PAGE, while the contents of 27 kD γ-zein and 14 kD β-zein proteins were not affected (Fig. 4b).

In plants, most respiratory chain related proteins are expressed by mitochondrial genome and undergo post-transcriptional processes regulated by nuclear genome expressed factors, including pentatricopeptide repeat (PPR) proteins. PPRs were reported to affect the endosperm, embryo and seedling development [28, 29]. We observed a significant developmental delay of the PPR/CAS9-9 and −13 plants compared to wild type at 35 days after pollination (35 DAP, Fig. 4c).

## Discussion

Maize is one of the most important cereal crops in the world. High efficient and accurate gene modification would benefit maize genetics study and breeding. We provided a framework to design, synthesize and use multiple tRNA-gRNA units for multiplex gene editing with CRISPR/Cas9 in maize (Figs. 1 and 2). These multiple tRNA-gRNA units were expressed under the control of the selected maize Pol III promoter (maize U6p). In this study, we successfully produced simultaneous mutagenesis of multiple genomic loci or deletion of short chromosomal fragments (Fig. 3). Our results showed that the optimized tRNA-processing system-based strategy is a robust and efficient tool for multiply targeted genome modification in maize. Our results also demonstrated that targeting one gene with two gRNAs using multiple tRNA-gRNA units greatly increased the efficiency of gene knock-out in maize. Compared to the parallel simplex editing system, the tRNA-processing strategy enables significantly higher editing efficiency (*p*-value = 0.021, Fig. 3). Given the extremely large number of tRNA genes and the fact that RNase P and RNase Z precisely recognize RNA substrates with tRNA-like structures [21, 30], there are many other choices of tRNA sequences to be embedded in the multiple tRNA-gRNA units in maize, implying higher efficiency of gene knock-out might be achieved with advanced design.

The mutation ratio of different construction for targeting different genes was varied, ranging from 57.1 to 71.4 % for simplex editing and 85.7–100 % for multiplex editing. There might be some factors regulating the mutation ratio, including the efficiency of gRNA to search and target to the specific site and different T-DNA insertion site in the genome [31]. Moreover, the mutation efficiency of CRIPSR/Cas9 system is variable in different plant species [32, 33]. The tRNA-processing system-based strategy enables the generation of many double-strand breaks (DSB) in genomic DNA. It may provide an efficient tool to help dissect the molecular process of chromosomal deletion. Due to the differences in the delivery, expression and activity of gRNAs and Cas9, it is not surprising to see some discrepancies in fragment-deletion frequency between stable transgenic plants containing different multiple tRNA-gRNA units (Fig. 3). Compared with RPL, the gRNA spacers selected for PPR were physically closer, approximately 200 bp apart in PPR and 300 bp apart in RPL, and had a higher sequence similarity, approximately 25 % similarity in PPR and 45 % similarity in RPL. The accurate chromosomal fragment deletion between two targets only existed in PPR/CAS9 lines but not in RPL/CAS9 lines (Fig. 3). Based on our results, we propose an improved sequence selection of gRNA spacers for high efficient chromosomal fragment deletion in which the distance between the two gRNA spacers should not be too long and the sequences of the two gRNA spacers should have high identity. gRNA targets of this type might have similar chromosome structure, binding ability, delivery and activity, causing generation of DSBs in the genome at the same time. The two reverse overlapping lncRNAs were targeted with a single 4 TGU construction that including two sites for lncRNA1 and two sites for lncRNA2. In plants transformed with this construct, a long-distance deletion beyond the sequence between the targets was observed. The way too high density and number of targets may have been the cause of this unintended mutation. As only SNPs at target sites were not useful for the knock-out of lncRNAs, long chromosomal fragment deletion fulfilled the complete abolishment of lncRNA function. Further more, it is also indicated that up to 4 TGUs design of tRNA-processing system-based strategy can still deliver gene modification in maize. Higher multiple TGUs design might also work in maize.

## Conclusions

This is the first report of successful multiplex gene editing using the tRNA-processing system in maize. This optimized tRNA-processing system-based strategy for maize can be broadly used for stable complete gene knock-out in the future. We propose that the tRNA-processing system-based strategy improves the efficiency of the CRISPR/Cas9 editing in maize. Additionally, advanced sequence selection of gRNA spacers to generate DSBs in the genome at the same time increases the efficiency and accuracy of chromosomal fragment deletions for complete abolishment of gene function especially lncRNAs, which is important for the enhancement of maize genetic research and breeding.

## Abbreviations

CRISPR/Cas9, clustered regularly interspersed short palindromic repeat/CRISPR-associated protein 9; gRNA, guide RNA; MT, multiple TGU; ORF, open reading frame; PAM, protospacer adjacent motif; TGU, tRNA-gRNA unit; U6p, U6 promoter; U6t, U6 terminator

## Additional file

Additional file 1: Table S1. The primers used in the experiments. (PDF 7 kb)
