# Supplementary material for: High-efficiency CRISPR/Cas9 multiplex gene editing using the glycine tRNA-processing system-based strategy in maize
Source: BMC Biotechnol. 2016 Aug 11;16:58. doi: 10.1186/s12896-016-0289-2 (PMC4982333; doi:10.1186/s12896-016-0289-2)
Supplement: Additional file 1: Table S1. — The primers used in the experiments. (PDF 7 kb) [file 12896_2016_289_MOESM1_ESM.pdf]

Table S1, Primers used in the experiments.

Usage	Name	Sequence
Vector construction	U6-P-5	5’-AAGTCGTAAAATAGTGGTGTC-3’
	U6-P-3	5’-AATTCGGTGCTTGCGGCTCG-3’
	U6-T-5	5’-GCGGATTCGCACATATGCCC-3’
	U6-T-3	5’-GCGAGGGCTAAATCGTTAAG-3’
Genotyping	BAR-5	5’-GAAGTCCAGCTGCCAGAAAC-3’
	BAR-3	5’-GCACCATCGTCAACCACTAC-3’
	MADS-5	5’-TTATGTTGCCTTGGTATTG-3’
	MADS-3	5’-TGCCGTCATCCATCTGTAA-3’
	MYBR-5	5’-CACACAGAATGGCATCGCCC-3’
	MYBR-3	5’-GACAGAAGAGTGTTCGAAGGCG-3’
	AP2-5	5’-GTATGTCATGCACAACACCTTCT-3’
	AP2-3	5’-TGGCGGTGAGACATTAGGAAG-3’
	RPL-5	5’-GTCGTTAACCCCGTCGATCC-3’
	RPL-3	5’-CGGACAGTGCCTCCTGAC-3’
	PPR-5	5’-ACAGATGCGATTGTGACTTGC-3’
	PPR-3	5’-TCCTGTCATCAGGAACCAAC-3’
	Lnc-5	5’-CGGGATCTCCACCTCTCTC-3’
	Lnc-3	5’-TCTCTCTGGATTCGCCGAC-3’
